# Serum cytokine profiles in healthy young and elderly population assessed using multiplexed bead-based immunoassays

**DOI:** 10.1186/1479-5876-9-113

**Published:** 2011-07-20

**Authors:** Hyun Ok Kim, Han-Soo Kim, Jong-Chan Youn, Eui-Cheol Shin, Sungha Park

**Affiliations:** 1 Department of Laboratory Medicine and Cell Therapy Center, Yonsei University College of Medicine, Seoul 120-752, Republic of Korea; 2 Division of Cardiology, Yonsei Cardiovascular Center, Yonsei University College of Medicine, Seoul 120-752, Republic of Korea; 3 Laboratory of Immunology and Infectious Diseases, Graduate School of Medical Science and Engineering, KAIST, Daejeon 305-732, Republic of Korea

## Abstract

**Background:**

Lipid metabolites and cytokines, including chemokines and growth factors, are the key regulators of immune cell function and differentiation, and thus, dysregulation of these regulators is associated with various human diseases. However, previous studies demonstrating a positive correlation of cytokine levels with aging may have been influenced by various environmental factors and underlying diseases. Also, data regarding cytokine profiling in the elderly are limited to a small subset of cytokines.

**Methods:**

We compared the profiles of 22 cytokines, including chemokines and growth factors, in a case-controlled study group of a gender-matched, healthy cohort of 55 patients over the age of 65 and 55 patients under the age of 45. Assessment of serum cytokine concentrations was performed using commercially-available multiplex bead-based sandwich immunoassays.

**Results:**

Soluble CD40 ligand (sCD40L) and transforming growth factor alpha (TGF-α) levels were significantly higher in the elderly patients, whereas granulocyte colony-stimulating factor (G-CSF), granulocyte-monocyte colony-stimulating factor (GM-CSF), and monocyte chemoattractant protein-1 (MCP-1) levels were significantly lower in the elderly patients. The partial correlation analysis demonstrating the correlation between cytokine levels when controlled for gender, systolic blood pressure, total cholesterol, HDL cholesterol, triglyceride, and serum creatinine levels further demonstrated that G-CSF, GM-CSF, and MCP-1 had significant negative correlations with age, whereas sCD40L and TGF-α had significant positive correlations.

**Conclusions:**

Future studies will focus on examining the significance of these age-related changes in circulating cytokines and other biological markers and their potential contribution to the development of different age-associated diseases.

## Background

Aging is accompanied by a decline in immune functions, referred to as immune aging or immune senescence. Paradoxically, life-long exposure to environmental factors and countless interactions with infectious agents leads to a chronic inflammatory state in older individuals, termed inflammaging, characterized by an increase in proinflammatory mediators present in serum [1,2]. Changes in T-cell homeostasis with aging are associated with a decline in immunity and increased inflammation. Increased accumulation of regulatory T cells contributes to impaired CD8 and natural killer cell activities [3,4]. Also, a decrease in naïve T cells may result in impaired acquired immune responses, whereas clonal expansion of CD25 null T cells may result in increased secretion of tumor necrosis factor-alpha (TNF-α) and interleukin-6 (IL-6), resulting in a heightened degree of inflammation [5].

Lipid metabolites and cytokines, including chemokines and growth factors, are the key regulators of immune cell function and differentiation. Thus, dysregulation of these regulators is associated with various human diseases. Age-associated elevation of inflammatory factors including TNF-α, IL-6, prostaglandin E_2 _(PGE_2_), and IL-1β have been described previously [6-8]. This elevation may be attributable to both the derangement of inflammation regulation and lifelong exposure of the immune system to environmental risk factors such as smoking, aging, hypertension, and diabetes [8-10]. However, previous studies that demonstrated positive correlations of cytokine levels with aging were performed in general aging populations that may have been influenced by various environmental factors and underlying diseases. Additionally, data regarding cytokine profiling in the elderly have been limited to a small subset of cytokines. In this study, we compared the profiles of 22 cytokines, chemokines, and growth factors in a case-controlled study group of a gender-matched, healthy cohort of 55 subjects over the age of 65 (Median age 68) and 55 subjects under the age of 45 (median age 34). The levels of the cytokines, chemokines, and growth factors were analyzed using multiplexed bead-based immunoassays.

## Methods

### Subject population

The study group was comprised of 110 healthy subjects who were enrolled in the Cardiovascular Genome Center (male:female = 44:66). The Cardiovascular Genome Center is a Korean government-sponsored research project with the objective of determining the genetic factors associated with the development of cardiovascular disease in a large, prospective study group. The study subjects were enrolled in the Cardiovascular Genome Center cohort as healthy control subjects. The study subjects did not have any past histories of hypertension, diabetes mellitus, cardiovascular disease, cerebrovascular disease, cancer, chronic renal disease, or any chronic inflammatory conditions. Group 1 consisted of 55 subjects under the age of 45 and group 2 consisted of 55 subjects over the age of 65. The study subjects were not permitted to perform strenuous exercise or drink alcoholic beverages 24 h before the laboratory test. The study subjects were also instructed to avoid eating or drinking anything except water during the testing period. Written, informed consent was obtained from all patients and the protocol was approved by the Institutional Review Board of Yonsei University College of Medicine (4-2001-0039). Research was conducted in compliance with the Helsinki Declaration.

### Blood collection

Blood samples were obtained from the forearm of each subject via the anticubital vein after a minimum of 12 hours of fasting. Samples were collected in EDTA-treated and plain tubes.

The methods for determining the concentrations of each lipid parameter were based on an enzymatic method (Hitachi 7600-110, Hitachi Co., Japan) that analyzed total cholesterol and triglyceride levels. After precipitation of serum chylomicron, LDL, and VLDL with dextran sulfate-magnesium, the HDL-C remaining in the supernatant fluid was measured using the enzymatic method (Hitachi 7600-110). LDL cholesterol levels were calculated using the Friedewald formula with serum triglyceride concentrations less than 4.52 mol/L (400 mg/mL) [11].

### Anthropometric and blood pressure measurements

The body weight and height of each undressed and barefoot subject were measured in the morning. After 5 minutes of rest, the brachial blood pressure was measured from the dominant arm using an OMRON HEM 7080 IT while the subject remained seated. The average of three measurements was recorded for each subject.

### Multiplex bead-based immunoassay

Simultaneous assessment of serum concentrations of epidermal growth factor (EGF), fibroblast growth factor 2 (FGF2), FMS-like tyrosine kinase 3 ligand (Flt-3L), granulocyte colony-stimulating factor (G-CSF), granulocyte-monocyte colony-stimulating factor (GM-CSF), interferon-α2 (IFN-α2), INF-γ, IL-10, IL-15, IL-17, IL-1β, IL-2, IL-6, IL-8, INF-γ inducible protein 10 (IP-10), monocyte chemoattractant protein-1 (MCP-1), macrophage inflammatory protein-1β (MIP-1 β), platelet-derived growth factor-AA (PDGF-AA), soluble CD40 ligand (sCD40L), transforming growth factor alpha (TGF-α), TNF-α, and vascular endothelial growth factor (VEGF) was performed using commercially-available multiplex bead-based sandwich immunoassay kits (MPXHCYTO-60K-25, Millipore, Billerica, MA) as per the manufacturer's instructions. Briefly, plasma samples (25 μL/well) or standards (25 μL well) were incubated with 25 μL of the pre-mixed bead sets in pre-wetted 96-well microtiter plates at 4°C overnight. After washing, 25 μL of the fluorescent detection antibody mixture was added for 30 min and 25 μL of streptavidin-phycoerythrin was added to each well for an additional 30 min at room temperature. A range of 3.2-10,000 pg/mL recombinant cytokines was used to establish standard curves and to maximize the sensitivity and dynamic range of the assay. Cytokine levels were determined using a Luminex IS 100 (Luminex, Austin, TX), and the data were reported as median fluorescent intensities.

### Statistical analysis

Results are expressed as means ± standard deviation. In this study, comparisons of discrete variables were made using the chi-square method and t-tests were used for continuous variables. Because the distribution of the cytokines was skewed, a log transformation of the cytokines was performed for independent t-tests and partial correlation analyses. For the partial correlation analysis, the correlation between aging and serum biomarkers was assessed while controlling for gender, smoking, body mass index (BMI), fasting blood glucose (FBG), systolic blood pressure (SBP), total cholesterol, HDL cholesterol (HDL), triglyceride (TG), and serum creatinine levels. A two-tailed value of P < 0.05 was considered statistically significant. All statistical analyses were performed using SPSS 13.0 (SPSS Inc., Chicago, IL).

## Results

**Figure 1:**
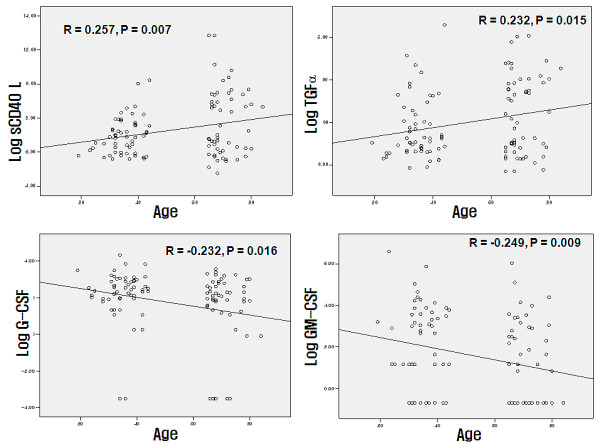
**Simple correlation between age and serum biomarkers (sCD40L, G-CSF, GM-CSF, and TGF-α in pg/mL)**. The × axis is age. The Y axis consists of log transformed sCD40L, G-CSF, GM-CSF and TGF-α. Simple correlation analysis was performed between age and the cytokines. Age showed significant positive correlation with log transformed sCD40L (R = 0.257, P = 0.007) and log transformed TGF-α (R = 0.232, P = 0.015), whereas age showed significant negative correlation with log transformed G-CSF (R = -0.232, P = 0.016) and log transformed GM-CSF (R = -0.249, P = 0.009).

Compared to the younger subjects in group 1, the elderly subjects in group 2 were associated with significantly higher SBP, total cholesterol, TG, serum albumin, serum blood urea nitrogen (BUN) and serum creatinine (Table 1). Comparison of the serum concentration of 22 cytokines-chemokines-growth factors demonstrated that sCD40L (group 2: 20370.6 ± 71662.0 pg/mL vs group 1: 2205.8 ± 4699.2 pg/mL, P value = 0.016) and TGF-α (group 2: 4.9 ± 4.8 pg/mL vs group 1: 3.2 ± 4.0 pg/mL, P value = 0.026) were significantly higher in the elderly subjects, whereas G-CSF (group 1: 14.7 ± 13.2 pg/mL vs group 2: 9.9 ± 8.8 pg/mL, P-value = 0.009), GM-CSF (group 1: 40.9 ± 108.6 pg/mL vs group 2: 20.3 ± 60.4 pg/mL, P value = 0.021) and MCP-1 (group 1: 213.5 ± 100.7 pg/mL vs group 2: 168.0 ± 73.0 pg/mL, P value = 0.027) were significantly lower in the elderly subjects (Table 2). The serum level of EGF, FGF-2, Flt-3L, INF-A2, INF-γ, IL-10, IL-15, IL-17, IL-1b, IL-2, IL-6, IL-8, IP-10, MIP-1β, PDGF-AA, TNF-α and VEGF showed no significant difference (Table 2). The partial correlation analysis demonstrating the correlation between cytokines-chemokines-growth factors when controlled for gender, SBP, total cholesterol, HDL, TG and serum creatinine demonstrated that G-CSF, GM-CSF and MCP-1 has a significant negative correlation with age whereas sCD40L and TGF-α has a significant, positive correlation (Table 3, Figure 1).

**Table 1:** Average baseline clinical characteristics of patients.

	Group 1 (Age < 45)	Group 2 (Age ≥ 65)	P-value^b^
**Gender (male:female)**	23:32	23:32	
**Age**	34.8 ± 5.7	70.4 ± 4.9	**< 0.001**
**SBP^b^(mmHg)**	115 ± 13	135 ± 19	**< 0.001**
**DBP (mmHg)**	75 ± 9	77 ± 11	0.383
**BMI (kg/m^2^)**	22.9 ± 3.6	23.3 ± 3.4	0.526
**Smoking (%)**	21 (38.2%)	6 (10.9%)	**0.001**
**T chol (mg/dL)**	192 ± 30	194 ± 35	**0.033**
**TG (mg/dL)**	110 ± 66	162 ± 122	**0.007**
**HDL (mg/dL)**	50 ± 12	51 ± 11	0.838
**FBG (mg/dL)**	84.8 ± 10.4	98.7 ± 38.3	**0.011**
**Albumin (g/dL)**	4.7 ± 0.3	4.5 ± 0.3	**< 0.001**
**BUN (mg/dL)**	11.7 ± 2.6	15.2 ± 3.8	**< 0.001**
**Cr (mg/dL)**	0.68 ± 0.17	0.79 ± 0.23	**0.007**

^a^Differences with P < 0.05 are considered significant.

^b^Abbreviations; SBP: Systolic blood pressure, DBP; diastolic blood pressure, BMI: body mass index, T chol: Total cholesterol, TG: Triglyceride, HDL: High density lipoprotein, FBS: Fasting blood glucose, BUN: Blood urea nitrogen, Cr: Creatinine

**Table 2:** Serum levels of cytokines, chemokines, and growth factors according to age.

Cytokines (pg/ml)	Group 1 (Age < 45)	Group 2 (Age ≥ 65)	P-value^a^
**G-CSF**	14.7 ± 13.2^b ^(0.03-75.8)	9.9 ± 8.8 (0.03-35.2)	**0.009**
**GM-CSF**	40.9 ± 108.6 (0.5-728.1)	20.3 ± 60.40 (0.50-415.1)	**0.021**
**MCP 1**	213.5 ± 100.7 (27.9-667.8)	168.0 ± 73.0 (39.34-355.9)	**0.027**
**sCD40L**	2205.8 ± 4699.2 (268.6-27703.8)	20370.6 ± 71662.0 (115.8-380396.7)	**0.016**
**TGF-α**	3.2 ± 4.0 (0.93-26.8)	4.9 ± 4.8 (0.86-20.8)	**0.026**
**EGF**	31.3 ± 35.9 (3.2-210.5)	61.0 ± 65.1 (3.20-251.6)	0.073
**FGF-2**	18.9 ± 11.3 (6.7-65.6)	20.1 ± 13.9 (3.20-72.83)	0.863
**Flt-3L**	10.2 ± 10.1 (0.84-59.3)	13.2 ± 15.9 (0.03-78.42)	0.759
**IFN-α2**	21.3 ± 22.6 (2.42-102.2)	33.3 ± 70.2 (2.42-449.2)	0.822
**IFN-γ**	13.1 ± 22.7 (0.14-126.8)	10.3 ± 18.4 (1.09-117.7)	0.948
**IL-10**	1.32 ± 3.06 (0.01-19.8)	1.58 ± 6.17 (0.01-41.7)	0.325
**IL-15**	3.04 ± 2.17 (1.25-13.1)	3.49 ± 4.31 (1.32-28.9)	0.668
**IL-17**	6.53 ± 7.42 (1.58-37.8)	12.2 ± 37.9 (1.43-275.1)	0.640
**IL-1β**	2.04 ± 4.93 (0.17-24.)	2.52 ± 7.41 (0.17-39.0)	0.645
**IL-2**	5.13 ± 2.31 (2.88-18.3)	5.58 ± 4.17 (3.06-32.1)	0.601
**IL-6**	2.91 ± 6.45 (0.16-37.7)	2.57 ± 5.22 (0.16-31.5)	0.750
**IL-8**	23.9 ± 29.7 (4.2-132.6)	27.6 ± 43.9 (4.76-217.0)	0.995
**IP-10**	462.2 ± 364.7 (145.3-2152.2)	451.3 ± 256.4 (149.8-1394.8)	0.673
**MIP-1β**	40.5 ± 38.8 (3.2-227.2)	40.4 ± 33.6 (3.20-231.1)	0.633
**PDGF-AA**	1528.3 ± 878.8 (140.6-3290.2)	1615.3 ± 1125.0 (55.3-3421.7)	0.485
**TNF-α**	3.21 ± 4.04 (0.93-26.8)	4.94 ± 4.79 (0.86-20.8)	0.916
**VEGF**	114.9 ± 147.1 (13.1-864.1)	100.5 ± 75.4 (6.9-329.3)	0.853

^a^Differences with P < 0.05 are considered significant.

^b^Average concentrations ± standard deviation in pg/mL.

**Table 3:** Partial correlation between aging and cytokines controlled for gender, smoking, body mass index, fasting blood glucose, SBP, total cholesterol, HDL, triglyceride, and creatinine levels.

	Correlation coefficient	P-value*
**EGF**	0.078	0.451
**G-CSF**	-0.214	0.037
**GM-CSF**	-0.297	0.003
**MCP-1**	-0.293	0.004
**Soluble CD40L**	0.277	0.007
**TGFα**	0.261	0.011

* P < 0.05 is considered significant.

## Discussion

To our knowledge, this is the first study that has compared extensive profiles of cytokines, including chemokines and growth factors, in healthy elderly and young subjects. As compared to the younger subjects in group 1, the elderly subjects had significantly higher SBP, total cholesterol, TG, serum albumin, serum blood urea nitrogen (BUN), and serum creatinine levels (Table 1). Comparison of the serum concentrations of 22 cytokines, chemokines, and growth factors demonstrated that sCD40L and TGF-α levels were significantly higher in the elderly subjects, whereas G-CSF, GM-CSF, and MCP-1 were significantly lower in the elderly subjects (Table 2). The serum levels of EGF, FGF-2, Flt-3L, INF-α2, INF-γ, IL-10, IL-15, IL-17, IL-1β, IL-2, IL-6, IL-8, IP-10, MIP-1β, PDGF-AA, TNF-α, and VEGF showed no significant differences between the two groups (Table 2). The partial correlation analysis demonstrating the correlation between the levels of the cytokines, chemokines, and growth factors when controlled for gender, SBP, total cholesterol, HDL, TG, and serum creatinine levels further indicated that G-CSF, GM-CSF, and MCP-1 had significant negative correlations with age, whereas sCD40L and TGF-α had significant positive correlations (Table 3 and Figure 1).

There was a lack of association of IL-6 levels with aging in the healthy study populations (Table 2), which is in concordance with previous studies [12,13]. However, unlike our findings that indicated no significant association of TNF-α, IL-6, and IL-1β levels with age, some previous studies have indicated that these cytokine levels are elevated in elderly subjects as compared to younger subjects [8,14-16]. A likely reason for the discrepancy is that in the previous studies, the elderly subjects were not controlled for associated diseases, such as hypertension and diabetes, which could increase inflammation. In a study by Ferrucci et al., controlling for cardiovascular risk factors attenuated the regression coefficient between aging and IL-6 [8]. In contrast to that study, we excluded subjects with previous histories of hypertension, cardiovascular disease, cerebrovascular disease, diabetes mellitus, cancer, or chronic renal disease, which minimized the confounding effects of concomitant disease processes that could alter the inflammatory state of the study patients. Additionally, in the study by Ferrucci et al., the highest level of IL-6 was in subjects over the age of 85, whereas the differences in IL-6 levels between subjects 65-74 years of age and patients 20-49 years of age was not as large [8]. The average age of the elderly subjects in this study was 70.4. Therefore, the lack of a very elderly population may be another possible explanation for the discrepancy in results.

This is the first study to demonstrate that sCD40L levels are significantly associated with aging (Tables 2 and 3 and Figure 1). The CD40/CD40L system belongs to the tumor necrosis factor superfamily and is a key pathway that links inflammation and atherothrombosis [17]. CD40 and CD40L are expressed in a variety of cell types, including platelets, vascular smooth muscle cells (VSMC), and immune cells [17,18]. Increased interactions between CD40 and CD40L may result in increased expression of cell adhesion molecules on endothelial cells and VSMCs, which subsequently results in increased vascular inflammation. Additionally, sCD40L and CD40 interactions increase oxidative stress and endothelial dysfunction, which may also contribute to an increase in the inflammatory cascade [17,19]. Increased secretion of sCD40L may be one explanation for the increased inflammation associated with aging, and may be a pathway that links aging with an increased risk of atherothrombosis.

TGF-α, a member of the EGF family, is a potent mitogen and chemotactic factor [20], and was positively correlated with aging (Tables 2 and 3 and Figure 1). TGF-α binds to the EGF receptor with a high affinity [21] and is indispensable for the proper development of many tissues and organs, wound healing, bone resorption, and angiogenesis [22]. TGF-α is implicated in numerous disease states, including coronary artery diseases, cystic fibrosis, psoriatic lesions, oral leukoplakia, submucosal fibrosis, Barrett's esophagus syndrome, and cancer [22]. Recent results also implicate this growth factor in the development of certain diabetic complications, such as atherosclerosis [23]. Though it is unknown whether TGF-α plays an important role in regulating homeostasis and/or diseases in aged subjects, increased serum levels of this cytokine in the elderly population may play a pathophysiological role in vascular remodeling and atherogenesis.

Monocytes and neutrophils, key components of the first line of defense, are the first inflammatory cells recruited to local tissue sites in response to infection or inflammation. Both G-CSF and GM-CSF are essential for leukocyte generation from hematopoietic stem cells, and are important mediators of the host response to infection. G-CSF and GM-CSF regulate other cell types in addition to neutrophils, such as monocytes, natural killer cells, and dendritic cells [24]. Like most growth factors and cytokines, G-CSF modulates cytokine profiles that alter the composition and function of immune cell populations. The serum levels of G-CSF and GM-CSF are often elevated in response to infection, suggesting that these hematopoietic cytokines play key roles in immunity [25]. The age-related decrease in circulating G-CSF and GM-CSF levels seen here may contribute to the impaired inflammatory responses and recruitment of leukocytes often seen in response to infections in elderly populations.

MCP-1 (CCL2), a member of the CC chemokine family, regulates monocyte migration by promoting their exit from the bone marrow into the circulation or from circulation to the site of inflammation [26,27]. An elevated baseline level of MCP-1 is associated with acute coronary syndromes [28]. However, the age-related decline of circulating MCP-1 seen in our study (Tables 2 and 3) is in sharp contrast to other reports that showed increased levels in aged populations [29,30]. This discrepancy may be due to the rigid selection criteria imposed in the current study to exclude patients with histories of hypertension, diabetes, or other disease-related conditions. The decreased production of G-CSF, GM-CSF, and possibly MCP-1 in the elderly population may partly explain the age related reduction of circulating monocytes and other leukocytes often observed in aged populations [31].

One of the limitations of the study is the fact that we could not match the percentage of smokers in the study population. However, we tried to minimize the influence of smoking on the levels of cytokines by controlling for smoking in the partial correlation analysis.

## Conclusions

Aging was associated with significant increases in the serum concentrations of sCD40L and TGF-α and significant decreases in the serum concentrations of G-CSF, GM-CSF, and MCP-1. Future studies will focus on understanding the significance of these age-related changes in circulating cytokines, chemokines, and other biological markers and their potential contribution to the development of various age-associated diseases.

## Competing interests

The authors declare that they have no competing interests.

## Authors' contributions

All authors participated in the study design, result interpretation and in the writing. HOK and HSK performed the analysis of the data and drafted the manuscript. JCY and ECS participated in the design of the study and performed the statistical analysis and SP conceived and designed the experiments and wrote the paper. All authors read and approved the final manuscript.
